# Evaluation of Blood Flow in a Reconstructed Gastric Conduit by Thermography in Esophageal Cancer Surgery

**DOI:** 10.70352/scrj.cr.24-0151

**Published:** 2025-07-01

**Authors:** Shuhei Ueno, Masahiro Kimura, Tsuyoshi Saito, Takahisa Hirokawa, Hirotaka Miyai, Ryo Ogawa, Shuji Takiguchi

**Affiliations:** 1 Department of Gastroenterological Surgery, Kariya Toyota General Hospital, Kariya, Aichi, Japan; 2 Department of Gastroenterological Surgery, Nagoya City University Graduate School of Medical Sciences, Nagoya, Aichi, Japan

**Keywords:** thermography, indocyanine green, gastric conduit, esophageal cancer

## Abstract

**INTRODUCTION:**

A complication of gastrointestinal anastomosis is anastomotic leakage; the incidence of anastomotic leakage following esophageal cancer surgery remains high. Several factors contribute to anastomotic leakage; however, blood flow to the reconstructed organ is the most significant factor. Currently, indocyanine green (ICG) fluorescence is widely used for evaluating blood flow; however, several issues have been observed, including allergic reactions to the drug. We investigated the usefulness of thermography (TG) for gastrointestinal blood flow evaluation.

**CASE PRESENTATION:**

Case 1 was a 76-year-old male who underwent thoracoscopic subtotal esophagectomy and gastric conduit reconstruction for esophageal cancer. ICG fluorescence was performed to evaluate gastrointestinal blood flow, and ICG fluorescence and TG were simultaneously performed. The early and final luminescent areas following ICG injection were consistent with the TG images. Case 2 was a 73-year-old male who underwent bypass surgery using a Y-shaped gastric conduit for esophageal cancer with pulmonary invasion. First, TG was simultaneously performed with ICG fluorescence following Y-shaped gastric conduit creation; subsequently, TG was performed again after the gastric conduit was placed via the subcutaneous route. As in Case 1, the TG images were consistent with the blood flow boundaries identified using ICG. Furthermore, the TG images, after the gastric conduit was placed in the neck region, showed blood flow boundaries.

**CONCLUSIONS:**

Although accumulation of similar cases is necessary, TG has the potential for use as an auxiliary diagnostic tool in clinical practice. Moreover, it is highly useful for indicating the possibility of reevaluation at short intervals, which is difficult to evaluate using ICG.

## Abbreviations

ICG
: indocyanine green
TG
: thermography

## INTRODUCTION

ICG fluorescence imaging is widely used in gastrointestinal surgery and is considered useful for reducing anastomotic leakage.^1,2)^ Conversely, the following issues with ICG have been observed:^3)^ (1) shock due to administered drugs, (2) inappropriate for repeated evaluations, and (3) expensive equipment. Additionally, ICG is not recommended for patients with an iodine allergy because it is an iodinated dye that fluoresces under infrared light.^3)^ Although the half-life of ICG is approximately 3 min, clinical experience and reports have indicated that it remains in the tissues for a relatively long time, making it difficult to reevaluate in a short period.^4)^ Although some models exist that are preloaded on endoscopes used in surgery, ICG testing is costly. TG has been used not only for industrial purposes but also in hospitals to determine body temperature. We here investigated the possibility of using TG for evaluating gastrointestinal blood flow, which is frequently used in esophageal surgery for reconstruction.

## CASE PRESENTATION

Case 1 was a 76-year-old male patient who underwent thoracoscopic subtotal esophagectomy for mid-thoracic esophageal cancer. Following abdominal manipulation, the stomach was pulled out through a small incision in the epigastrium to create a gastric conduit that was approximately 4-cm-wide ICG (Daignogreen; Dai-Ichi Pharm, Tokyo, Japan) was injected as a bolus (0.25 mg/kg) from the peripheral vein to evaluate blood flow. Furthermore, we used a T530 thermal camera (FLIR Systems, Tokyo, Japan) and analysis software (ResearchIRMAX; FLIR Systems, Tokyo, Japan) to evaluate blood flow.

**Fig. 1:**
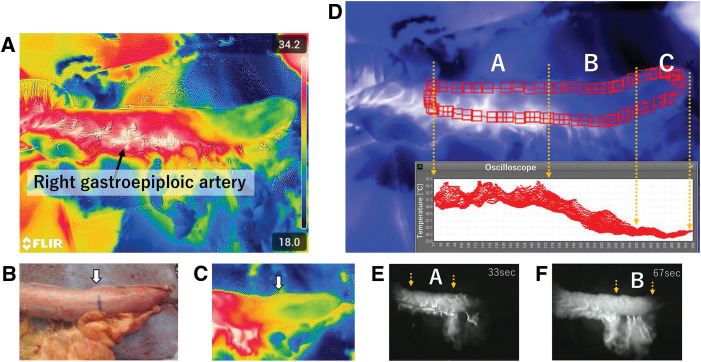
(**A**–**D**) TG images of the entire gastric conduit. (**E**, **F**) ICG images of the corresponding part of the gastric conduit. (**A**) The right gastroepiploic artery shows the highest temperature and appears white; (**B**) visual inspection (arrow); (**C**) TG image showing a change in the color tone at the boundary obtained using ICG (arrow); and (**D**) the upper panel shows the surface temperature analysis of along the entire length of the gastrointestinal tract using TG. The lower panel indicates that region A, which emits early on ICG, shows a high temperature in TG, whereas the region B that emits later on ICG shows a gradual decrease in surface temperature in TG over time; the region C that does not emit on ICG shows a low-temperature state in TG. (**E**) Luminous area (region A) 33 seconds after ICG injection and (**F**) luminous area (region B) 67 seconds after ICG injection. ICG, indocyanine green; TG, thermography

At approximately 30 s after ICG injection, the gastroepiploic arteries and arterioles within the gastric wall began fluorescing; gradually, the entire gastric wall began to fluoresce. At 67 s later, the area emitting fluorescence was fixed. Subsequently, TG imaging was performed from the field, wherein the gastroepiploic artery showed a hotter color and the gastric wall a slightly cooler color (**Fig. 1A** and **1C**). TG images revealed changes in surface temperature at the site of marking (**Fig. 1B**). Using the provided software, we further analyzed the TG images. The stomach wall, which showed early emission by ICG, exhibited a relatively constant temperature range of 30°C–34°C by TG, whereas the temperature on the oral side showed a gradual decrease to approximately 26°C (**Fig. 1D**). The stomach wall, which did not emit fluorescence by ICG, reached a plateau at a lower temperature (**Fig. 1E**). On the oral side, the surface temperature reached a plateau at lower temperatures (**Fig. 1F**). Changes over time in the gastrointestinal tract emitted by ICG were similar to the TG imaging and analysis results.

**Fig. 2:**
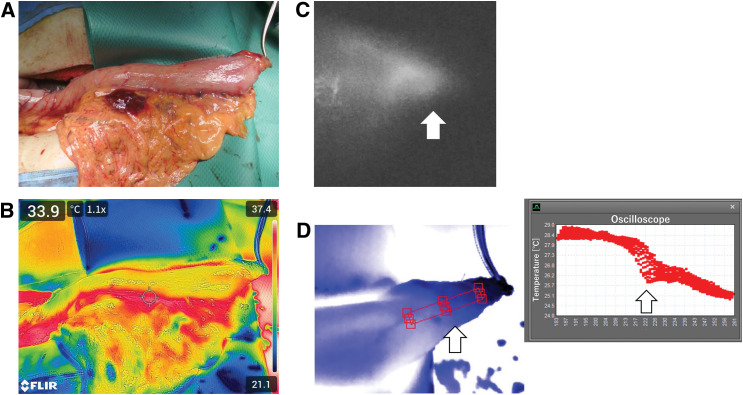
(**A**–**D**) Evaluation of blood flow in the gastric conduit in Y-shaped bypass surgery. (**A**) Visual inspection; (**B**) TG image of the gastric conduit; (**C**) ICG image of the apical portion of the gastric conduit. Arrow indicate the leading edge where luminescence is observed; and (**D**) TG images and analysis results, showing a steep temperature drop (arrow) at the site determined to be the blood flow boundary by ICG. ICG, indocyanine green; TG, thermography

**Fig. 3:**
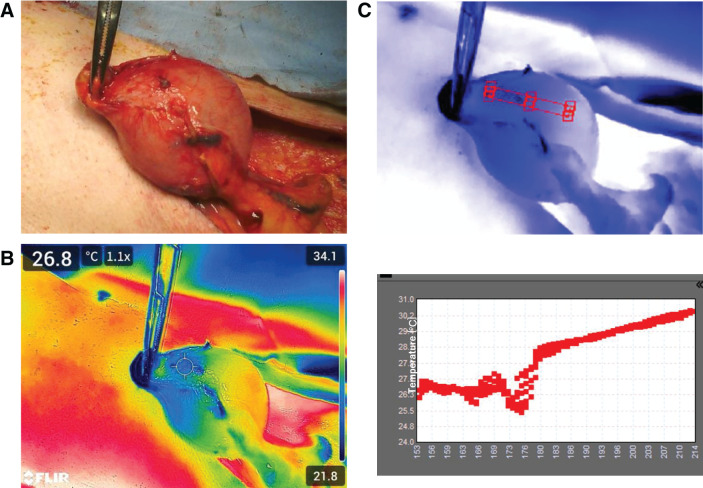
(**A**–**C**) Evaluation of blood flow in the gastric conduit elevated in the neck. (**A**) Visual inspection; (**B**) TG image showing a decreased surface temperature at the tip; and (**C**) analysis of TG images. A steep temperature drop is noted at the tip of the gastrointestinal tract. TG, thermography

Case 2 was a 73-year-old male patient who underwent bypass surgery using a Y-shaped gastric conduit for pulmonary invasion of esophageal cancer. The surgery was performed through an upper midline abdominal incision. A small hole was created on the pylorus using a circular stapler, and a 4-cm-wide greater curvature side gastric conduit was created using a linear stapler. To evaluate blood flow, we used ICG and TG. Although identifying blood flow boundaries indicated by ICG in a normal TG image is difficult (**Fig. 2B**), the analyzed oscilloscope (**Fig. 2D**) showed a sharp temperature decrease around the blood flow boundaries identified by ICG. After elevating the gastric conduit through the subcutaneous route to the neck, TG images were taken again (**Fig. 3A** and **3B**). Although determining blood flow boundaries by visual inspection was challenging, TG images revealed a decreased surface temperature at the tip. The oscilloscope that analyzed the TG images of the tip of the gastrointestinal tract showed a decreased surface temperature and a plateau change (**Fig. 3C**), which are similar to the changes obtained in Case 1. We determined this boundary as the blood flow boundary; following esophagogastric conduit anastomosis, we resected the area where the temperature change was observed.

Both patients had a good postoperative course with no postoperative anastomotic leakage or stenosis.

## DISCUSSION

In gastrointestinal surgery, ICG fluorescence imaging is widely used and is considered useful for reducing anastomotic leakage.^1,2)^ The advantages of ICG fluorescent staining include the ability to objectively evaluate the gastrointestinal blood flow by observing through an infrared camera and a short half-life of approximately 3 min. The disadvantages of ICG fluorescent staining include the cost of introducing the infrared camera system and the reported cases of patients with allergies to ICG.^3)^ Despite the short half-life of ICG, it is metabolized in the liver, making the objective judgment of blood flow difficult. The normal ICG 15-min retention rate, which is also used for evaluating liver function, is 18%–20%, and 20% remains in the blood even after 15 min.^4)^

Furthermore, we have been using ICG not only in the gastrointestinal tract but also in the small and large intestines and liver, and we were looking for an evaluation method that could assist or substitute for ICG.^1)^ TG is used for body temperature measurement in hospitals as well as in industrial applications, so we believe that it can be used for blood flow assessment.

**Fig. 4:**
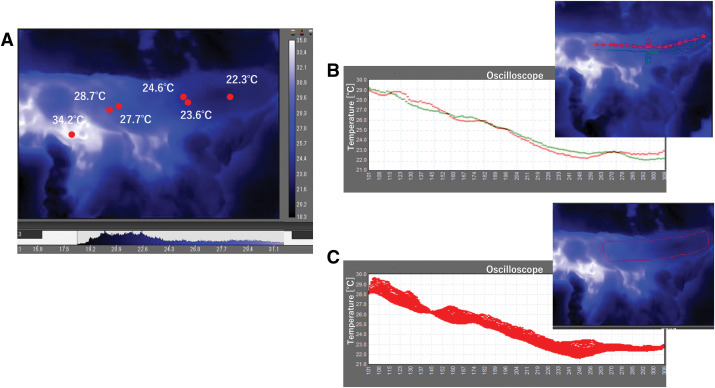
(**A**)–(**C**) Surface temperature of the gastric conduit measured using TG. (**A**) Measured value by point; (**B**) measured by line; and (**C**) measured by surface. TG, thermography

Some studies using TG for blood flow evaluation are scattered.^5–9)^ However, these studies compare the surface temperature at 1 point of an organ with that at a 2nd point. The TG images showed that the surface temperature of the gastric conduit was lower at the mouth of the tube (**Fig. 4A**). However, differences in surface temperatures in the immediate vicinity of a single point could be observed, sometimes by >1°C, depending on the site. Therefore, evaluating the gastrointestinal blood flow along a line, and even over a surface, rather than comparing 2 points, is significant (**Fig. 4B** and **4C**). In our cases, the analysis software provides a selected range of temperatures, and the oscilloscope shows the abrupt changes in temperature as a boundary of blood flow. This is clearly different in accuracy from the surface temperature and 2-point comparisons that have been reported using TG for blood flow evaluation. Since September 2021, we have conducted basic experiments with TG using porcine small intestine, and after obtaining Ethics Committee approval, we have conducted comparative studies of TG and ICG fluorescence in various organs in approximately 150 cases. Evaluating blood flow in the gastric conduit in esophageal cancer was performed in 12 cases. The presence of intramural blood flow, which is widely recognized in the stomach, is the major difference between the gastrointestinal tract and the large and small intestines in blood flow evaluation by TG. Therefore, when ICG fluorescence imaging is performed, the right gastroepiploic artery is visualized 1st, followed by the gastric wall in the same region, and then sequentially the tip of the gastrointestinal tube. We believe that the results of the TG analysis in the present cases are valuable because they represent these changes over time on an oscilloscope.

The usefulness of TG compared with ICG in evaluating blood flow in the esophageal reconstructed gastric conduit was evaluated. Although the cases did not develop allergies, the test proved to be noncontact and repeatable without the need for drug administration. Furthermore, TG and the analysis software are relatively inexpensive equipment. However, the influence of room temperature and other factors cannot be neglected. We aimed to establish a more effective blood flow evaluation method using TG by accumulating more cases.

## CONCLUSIONS

Although further case accumulation is needed, TG has potential as an auxiliary diagnostic tool for evaluating blood flow within the gastric conduit after esophageal reconstruction. In addition, TG is highly useful for indicating the possibility of reevaluation at short intervals, which is difficult to assess with ICG. Moreover, TG is considered safe because it does not require the administration of drugs and there is no risk of allergic reactions.

## DECLARATIONS

### Funding

This work was supported by JSPS Grant-in-Aid for Scientific Research C (Grant Number 22K08831).

### Authors’ contributions

S.U.: conceptualization, data curation, and writing the original draft.

M.K.: conceptualization, review, and editing of the draft.

T.S., T.H., H.M., R.O.: review and editing of the draft.

S.T.: supervision, review, and editing of the draft.

All authors have reviewed and approved the final manuscript, and each author agrees to take responsibility for all aspects of the study.

### Availability of data and materials

The data used in this study are available from the corresponding author upon reasonable request.

### Ethics approval and consent to participate

This study protocol was approved by the Institutional Review Board of Nagoya City University (60-22-0090). We also explained to the patients that TG would be used as an adjunctive examination to ICG and obtained their consent to participate in the study.

### Consent for publication

A written informed consent for publication was obtained directly from the patient.

### Competing interests

The authors declare that they do not have any conflicts of interest regarding the publication of this article.
